# Carbon Dioxide Sensing Modulates Lifespan and Physiology in *Drosophila*

**DOI:** 10.1371/journal.pbio.1000356

**Published:** 2010-04-20

**Authors:** Peter C. Poon, Tsung-Han Kuo, Nancy J. Linford, Gregg Roman, Scott D. Pletcher

**Affiliations:** 1 Huffington Center on Aging, Baylor College of Medicine, Houston, Texas, United States of America; 2 Department of Molecular and Human Genetics, Baylor College of Medicine, Houston, Texas, United States of America; 3 Department of Molecular and Integrative Physiology and Geriatrics Center, University of Michigan, Ann Arbor, Michigan, United States of America; 4 Biology and Biochemistry Department, University of Houston, Houston, Texas, United States of America; The Salk Institute for Biological Studies, United States of America

## Abstract

For nearly all life forms, perceptual systems provide access to a host of environmental cues, including the availability of food and mates as well as the presence of disease and predators. Presumably, individuals use this information to assess the current and future states of the environment and to enact appropriate developmental, behavioral, and regulatory decisions. Recent work using the nematode worm, *Caenorhabditis elegans*, and the fruit fly, *Drosophila melanogaster*, has established that aging is subject to modulation through neurosensory systems and that this regulation is evolutionarily conserved. To date, sensory manipulations shown to impact *Drosophila* aging have involved general loss of function or manipulation of complex stimuli. We therefore know little about the specific inputs, sensors, or associated neural circuits that affect these life and death decisions. We find that a specialized population of olfactory neurons that express receptor Gr63a (a component of the olfactory receptor for gaseous phase CO_2_) affects fly lifespan and physiology. Gr63a loss of function leads to extended lifespan, increased fat deposition, and enhanced resistance to some (but not all) environmental stresses. Furthermore, we find that the reduced lifespan that accompanies exposure to odors from live yeast is dependent on Gr63a. Together these data implicate a specific sensory cue (CO_2_) and its associated receptor as having the ability to modulate fly lifespan and alter organism stress response and physiology. Because Gr63a is expressed in a well-defined population of neurons, future work may now be directed at dissecting more complex neurosensory and neuroendocrine circuits that modulate aging in *Drosophila*.

## Abstract 2

Loss of an olfactory receptor that senses carbon dioxide increases longevity and fecundity and alters overall physiology in fruit flies.

## Author Summary

Sensory inputs, including taste and smell, can modulate lifespan in organisms such as fruit flies and nematodes. For example, the smell of live yeast is sufficient to accelerate aging in fruit flies that are nutrient restricted. However, the sensory pathways and specific olfactory cues that modulate aging are unknown. Here, we show that the olfactory receptor for carbon dioxide, Gustatory Receptor 63a (Gr63a), plays a role in determining longevity in *Drosophila melanogaster*. Flies lacking Gr63a function live longer, have increased fat storage, and exhibit greater reproductive output than control flies. Ablation of the neurons that express Gr63a also results in long-lived flies. Notably, the smell of live yeast does not affect the lifespan of flies that lack a functional Gr63a receptor, which suggests that carbon dioxide is a key regulatory molecule of this complex odor. Because Gr63a expression is restricted to a very select population of neurons, these results implicate a specific neural circuit in the modulation of fly lifespan.

## Introduction

The sensory neurons that constitute perceptual systems trigger countless decisions on a daily basis. By providing a viewport to the world, neurosensory systems help individuals to assess the current and future states of their surroundings and to enact appropriate responses. Whether to consume food, accept a mate, or simply move in a particular direction is influenced by inputs involving senses such as sight, taste, and smell. Such overt behavioral decisions can have obvious consequences: the proper identification of nutritious food might help you live another day, whereas the inability to correctly recognize a predator could mean you *become* food for one better equipped.

Not all sensory-mediated responses are so easily identified. Chemical cues associated with predation, fear, or anxiety may evoke a chronic stress response in rats involving subtle activation of the neuroendocrine stress axis and changes in central nervous system limbic circuitry [1],[2]. In mice, small populations of neurons in the hypothalamus may integrate metabolic and sexual odorant cues to initiate changes that foster reproduction [3]. Even highly conserved processes that were thought to be regulated predominantly at the cellular level may depend on sensory input. In the nematode worm, *Caenorhabditis elegans*, the molecular response to misfolded proteins, which has traditionally been studied as a cell-autonomous process, is strongly modulated by general neuronal signaling [4]. A second putative cell-autonomous response in the worm, the heat shock response, depends on a single thermosensory neuron, which is responsible for sensing ambient temperature [5].

Perhaps most remarkable has been the discovery that aging itself can be subject to neurosensory regulation. Indeed, such regulation has been shown to be evolutionarily conserved and important for modulating lifespan in both *C. elegans*, and the fruit fly, *Drosophila melanogaster*. Ablation of key sensory neurons in the nematode or mutations that result in their loss of function can modulate lifespan, with some neurons limiting lifespan and others promoting it [6],[7]. In the fruit fly, *D. melanogaster*, a loss-of-function mutation in Odorant Receptor 83b (Or83b), which broadly reduces olfactory capabilities, produces a significant increase in stress resistance and lifespan [8]. Fat deposition is also altered in these mutant flies, suggesting that olfaction may impact global energy balance [8]. Furthermore, expression of the transcription factor skn-1 in two sensory neurons in *C. elegans* is required for reduced nutrient availability (also called *dietary restriction*, which increases longevity) to affect nematode lifespan, albeit that this effect seems to be independent of environmental sensing [9]. Even temperature may modulate worm aging at least partially through its activation of thermosensory neurons [10].

What are the sensory cues and neural circuits that activate longevity programs? These remain largely unknown. There are, however, a few clues. Exposing *Drosophila* to food-derived odorants (odors from live yeast in particular) reduces lifespan when flies are confined to conditions of dietary restriction [8]. This effect is absent when they are fully fed, suggesting that the perception of high nutrient availability is sufficient to reverse some of the benefits of diet restriction. Similarly, in *C. elegans*, lifespan extension from bacterial deprivation is partially suppressed by a diffusible component of the bacterial food source [11]. Crude pheromone extracts have also been reported to impact nematode lifespan [12], but the active component is still in question [7],[12]. The identification of specific sensory cues and their receptors that modulate lifespan would help decipher the “sensory code” that modulates conserved mechanisms of physiology and aging. Because signals that originate from the sensory structures must be communicated to distant tissues to coordinate pro-longevity events, it would also facilitate the dissection of neurosensory and neuroendocrine circuits that specify longevity programs as well as the identification of molecular signaling pathways in distal tissues that enact them.

Here, we establish that a specialized population of olfactory neurons modulates the lifespan and physiology of *D. melanogaster*. In particular, we find that loss of function in Gustatory Receptor 63a (Gr63a), which serves as a component of the olfactory receptor for gaseous phase CO_2_, extends fly longevity and enhances fat deposition. Extended lifespan is accompanied by increased reproductive output and enhanced resistance to some (but not all) environmental stresses. Unlike previous reports involving more general olfactory manipulations, extended longevity via loss of Gr63a occurs through a mechanism that is likely independent of dietary restriction. We do, however, find that Gr63a is required for odorants from live yeast to affect longevity, suggesting that with respect to lifespan, CO_2_ is an active component of this complex odor. Because Gr63a is expressed in a highly specific population of CO_2_-sensing neurons (the ab1C neurons) that innervate a single glomerulus in the antennal lobe (the V glomerulus), these data implicate a specific sensory cue and its associated neurosensory circuit as having the ability to modulate fly lifespan and alter organismal stress response and physiology. Our results set the stage for the dissection of more complex neurosensory and neuroendocrine circuits that modulate aging in *Drosophila*.

## Results

Carbon dioxide is a ubiquitous ecological stimulus that has been shown to play important roles in insect ecology. Honeybees initiate behaviors that ventilate the hive in response to high CO_2_ levels. Blood-feeding insects use CO_2_ to track down hosts [13], and herbivorous insects use CO_2_ to detect desirable leaves, flowers, or fruits [14]. In *D. melanogaster*, sensory perception of CO_2_ may provide information on the availability of food sources; living and rotting fruit tissue produce CO_2_, as does respiration in live yeast. Flies are generally repelled by levels of CO_2_ as low as 0.1% above ambient, however; perhaps as a result of its postulated role as a component of an avoidance pheromone released by stressed adults [15]. Based on a possible dual role in mediating perception of food and stress—both of which are known modulators of aging—we decided to test the hypothesis that systems involved in the detection of CO_2_ modulate lifespan and physiology in the adult fly.

### The CO_2_ Olfactory Receptor Modulates Lifespan in *Drosophila*

*Drosophila* detect CO_2_ from the atmosphere using a small subpopulation of sensory neurons that are located in the antennae and that have been designated ab1C [16]. These neurons are required for CO_2_ avoidance behavior, and they express at least two gustatory receptor genes, *Gr63a* and *Gr21a*, which together comprise a CO_2_ odorant receptor [17]–[19]. They innervate a single, ventrally located glomerulus (the V glomerulus), which responds selectively to CO_2_ [18].

**Figure 1:**
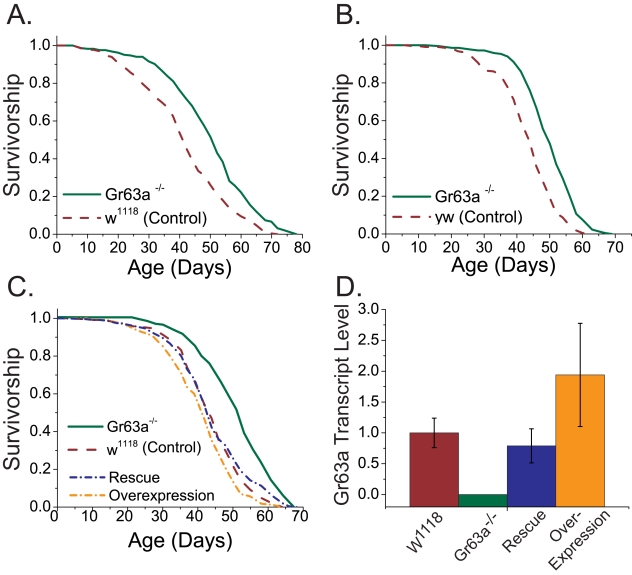
Flies with loss of function in Gr63a are long lived. (A and B) Survivorship of female flies homozygous for *Gr63a^1^* loss-of-function (null) allele (green solid lines) and their respective background control (red dashed lines) (A) *w^1118^* background (*n* = 249 and 254 for mutant and control flies, respectively), (B) *yw* background (*n* = 219 and 216 for mutant and control flies, respectively); *p*<1×10^−15^ in both cases. (C) Survivorship of flies from a transgenic rescue experiment. Expression of *Gr63a* cDNA under the control of its endogenous promoter region using the UAS/GAL4 system restored the natural lifespan of *Gr63a^1^* mutant flies (compare green solid lines to blue dot-dash line and red dashed line; *p* = 0.15), whereas overexpression of *Gr63a* had modest negative effect on lifespan (yellow dot dashed line vs. red dashed lines; *p* = 0.005). Samples sizes are as follows: control, 230; *Gr63a* ^−/−^, 228; overexpression, 230; rescue, 240. (D) Quantitative real-time PCR using RNA extracted from fly heads revealed that *Gr63a^1^* mutant flies lacked measureable *Gr63a* mRNA (no amplification was observed). Furthermore, transgenic rescue restored *Gr63a* mRNA to levels that were indistinguishable from *w^1118^* control (*p* = 0.59), and overexpression resulted in a roughly 2-fold increase in *Gr63a* transcript (but this was not significant; *p* = 0.32). Forty fly heads were used per RNA extraction, and expression measures were obtained from multiple quantitative PCR reactions each from at least three independent replicate RNA extractions. Error bars represent standard errors of the mean.

We obtained flies that lack a functional CO_2_ receptor due to a null mutant allele of *Gr63a* (*Gr63a^1^*; see also [18]). To insure proper control of genetic background, which has a significant potential to confound longevity studies, we backcrossed the *Gr63a* mutant flies at least ten generations to two independent, genetically heterogeneous laboratory stocks (*yw* and *w^1118^*). *Gr63a^1^* mutant ab1C neurons have been shown to lack an electrophysiological response to CO_2_, and mutant flies fail to show a wild-type avoidance behavior when exposed to CO_2_ in a T-maze [18]. Following backcrossing, we therefore confirmed the loss of Gr63a transcript (Figure 1D) and CO_2_ avoidance response in the backcrossed mutant lines (Figure S1).

To determine whether a lack of CO_2_ sensing affected longevity, we measured the lifespans of male and female *Gr63a^1^* mutant flies. We controlled for maternal effects and differences in larval environment by comparing sibling homozygous mutant animals and wild-type control animals from a cross of heterozygous parents. We found that female mutant flies exhibited a significantly longer lifespan (up to 30%) than their sibling controls in both *w^1118^* and *yw* genetic backgrounds (Figure 1A, 1B, and Table S1). Surprisingly, lifespan extension was observed only for females; male longevity was unaffected by the mutation (Figure S2A, Table S1). As further evidence for the *Gr63a* mutant effect, we measured longevity in the *w^1118^* background after an additional ten generations of backcrossing (for a total of 20) and found a comparable longevity extension (Figure S2B, Table S1). Gr63a loss of function increases lifespan predominantly by a reduction in the baseline mortality rate, rather than a change in the slope of mortality (Figure S2C) [8],[20]. These data suggest that loss of function of Gr63a extends lifespan in a sex-specific manner and that this lifespan extension is robust to variability in genetic background.

To further establish a connection between CO_2_ sensing and longevity, we measured the lifespans of heterozygous flies carrying one copy of the *Gr63a^1^* mutation and one wild-type allele. Published data suggest that, with regard to the function of CO_2_ sensing, the *Gr63a^1^* null allele is recessive [18]. We would therefore predict this to be true of longevity as well. To protect against a confounding of the results by hybrid vigor, we first backcrossed the *Gr63a^1^* mutation for 12 generations into the *w^1118^* background. We then allowed flies with both wild-type and mutant alleles to intermix in an unselected population for another six generations (total 18 generations). Eggs were collected from parents of mixed genotype and aliquoted in equal numbers to fly bottles for rearing. Siblings were then separated based on eye color as either wild-type, heterozygous, or homozygous for the *Gr63a* mutation. We found that, despite extreme genetic and environmental homogeneity, the *Gr63a* homozygous mutant flies lived longer than both wild-type (*p* = 4×10^−5^) and heterozygous animals (*p* = 1×10^−7^), whereas the lifespan of heterozygous flies was indistinguishable from that of wild-type flies (*p* = 0.24) (Figure S3A). *Gr63a^1^* is therefore recessive with respect to longevity extension, as it is for CO_2_ sensing.

### Transgenic Expression of Gr63a Rescues Longevity in *Gr63a^1^* Mutants

To ensure that the longevity extension observed in *Gr63a^1^* mutant females was due to the *Gr63a* mutation itself, we executed a genetic rescue using the Gal4/UAS bipartite system [21]. A fly line carrying a transgenic construct that allowed the GAL4 transactivator to be expressed under the control of the native *Gr63a* enhancer/promoter elements (*Gr63a*-GAL4 flies [18]) was backcrossed to the *w^1118^* background for seven generations. This was repeated with flies that carried a copy of *Gr63a* coding sequence under the control of the yeast upstream activating sequence (UAS-*Gr63a* flies; [18]). mRNA expression of *Gr63a* was rescued to wild-type levels in *Gr63a^1^* mutant flies that carried a copy of both transgenes. Although wild-type flies with a copy of both the GAL4 and UAS constructs appeared to exhibit roughly 2-fold enhanced expression of *Gr63a*, the data were variable and differences were not statistically significant (*p* = 0.32, Figure 1D). We found that transgenic expression of *Gr63a* restored mutant longevity back to wild-type levels (Figure 1C), which provides strong evidence that longevity extension in these flies is due to Gr63a loss of function. Overexpression of *Gr63a* did not significantly alter lifespan (Figure 1C).

### Genetic Ablation of ab1C Neurons Phenocopies the Longevity Extension of a *Gr63a* Loss-of-Function Mutation

**Figure 2:**
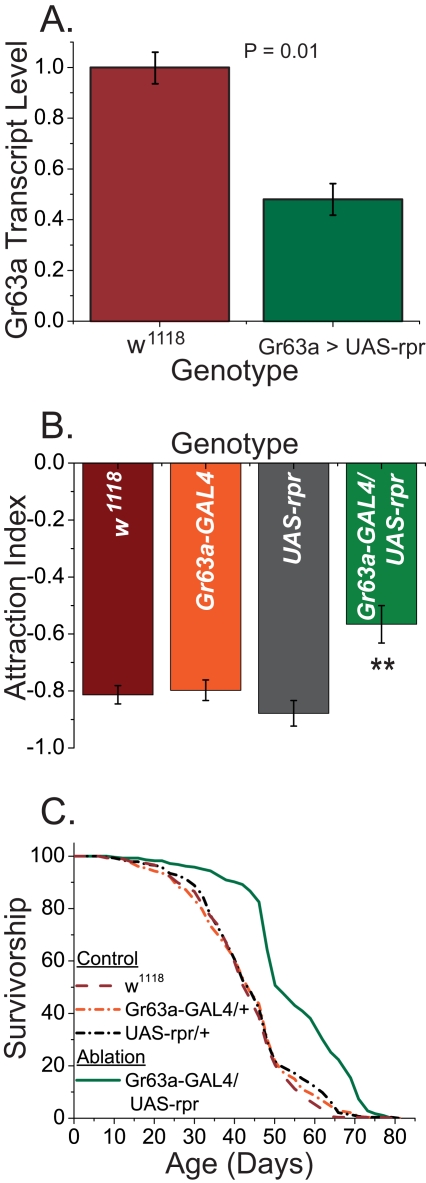
Genetic ablation of *Gr63a*-expressing cells phenocopies *Gr63a* mutant longevity. To further establish a role for *Gr63a*-expressing neurons in the modulation of lifespan, we expressed the proapoptotic gene reaper using *GR63a*-GAL4 to ablate the associated neurons. (A) *Gr63a* mRNA is reduced in the heads of female flies with both *Gr63a*-GAL4 and UAS-reaper compared to *w^1118^* control (*p* = 0.01). Expression was determined using at least three independent RNA extractions of 40 fly heads each. (B) *Gr63a*>reaper flies are significantly less repulsed by CO_2_ compared to control flies, UAS-reaper only, and *Gr63a*-GAL4 only (*p* = 0.002, 0.0007, and 0.003 respectively). Data were obtained using a T-maze assay and a stream of ∼4% CO_2_. Forty flies were used for each measure, and a minimum of eight measures were made for each genotype. (C) Female *Gr63a*>reaper flies (green solid line) are longer lived than corresponding controls, including *w^1118^* wild type (red dashed line), UAS-reaper only (dark gray dashed line), and *Gr63a*-Gal4 only (orange dashed line) control (*p*<1×10^−15^ for comparing *Gr63a*>reaper to any control line). There are no significant differences among the survivorship curves from the three control lines (*p*>0.05). Samples sizes are w1118, 292; GAL4/UAS, 286; UAS only, 282; GAL4 only, 286. Error bars represent standard errors of the mean.

To further verify that the lifespan extension of *Gr63a^1^* mutants was due to loss of neuronal function, we used the *Gr63a*-GAL4 lines to express the proapoptotic gene reaper (UAS-rpr) to ablate the ab1C neurons. Levels of *Gr63a* transcript were significantly reduced in *Gr63a*-GAL4;UAS-rpr lines compared to controls (Figure 2A), and neuron-ablated flies exhibited significantly reduced avoidance to ∼4% CO_2_ when tested in a T-maze (Figure 2B, *p* = 0.002). Notably, *Gr63a*-GAL4;UAS-rpr flies retained significant CO_2_-sensing capabilities, suggesting that the ablation was not complete. Nevertheless, we asked whether this degree of loss of function was sufficient to impact lifespan of female flies. We found that neuron-ablated flies experienced a significant increase in longevity over all wild-type and single-construct control lines and that the magnitude of longevity extension was comparable to that seen in *Gr63a^1^* homozygous mutant animals (Figure 2C). As with the mutants, increased lifespan resulted primarily from a change in the initial mortality rate (Figure S4). These data suggest that partial loss of ab1C neurons is sufficient to impact CO_2_ perception and increase fly lifespan.

### Loss of CO_2_ Sensing Alters Physiology and Stress Response in *Drosophila*

Fat metabolism, reproduction, and stress response are often linked with aging at the phenotypic level, yet the molecular mechanisms by which they are coupled remain poorly understood. Aging is associated with obesity, reproductive cessation, and chronic stress; and manipulations that extend lifespan often decrease reproductive output while enhancing fat deposition and stress response [22],[23]. In certain cases, however, the phenotypes can be uncoupled, and these instances provide insight into their underlying mechanisms [24].

**Figure 3:**
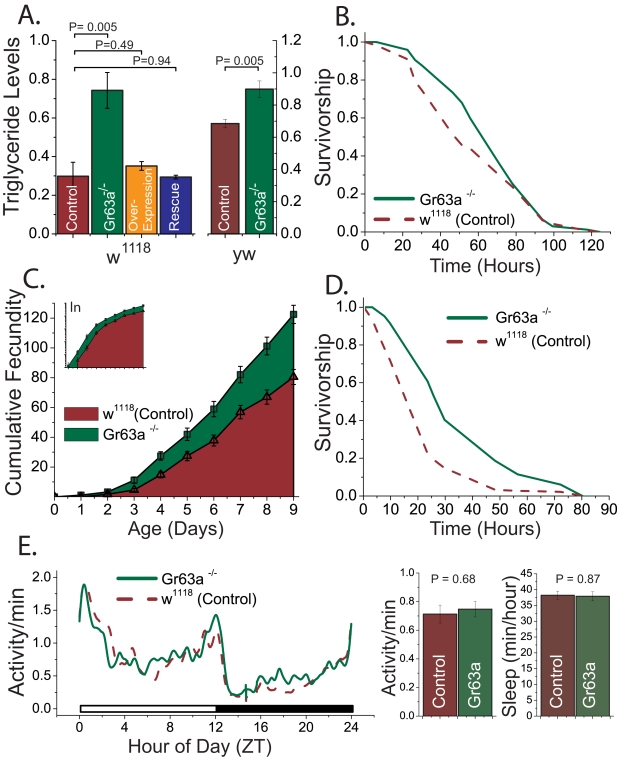
Flies with loss of function in Gr63a exhibit alterations in physiology and changes in resistance to particular stresses. (A) Female *Gr63a^1^* mutants exhibited higher triglyceride levels than control flies. This was observed in two independent genetic backgrounds, *w^1118^* and *yw*. Transgenic rescue of Gr63a restored mutant fat levels to that seen in wild-type flies. Overexpression of *Gr63a* had no effect on triglyceride levels. For each triglyceride measure, six independent measures of five females each were obtained and measured for triglyceride content. Data were normalized to total protein levels. (B) *Gr63a^1^* mutant flies (solid green lines) had normal starvation resistance compared to controls (dashed red lines) when placed on 1% agar, despite higher triglyceride levels shown in (A) (*p* = 0.10). (C) Over the course of 10 d, *Gr63a* mutants (green) laid roughly 40% more eggs than controls (red). Fifteen 2-d-old virgin females were each mated with two control males and subsequently maintained on 15% yeast-sucrose diet. Flies were moved to new vials daily, and eggs were counted each day for 10 d. The plot is representative of three independent experiments (*p*<0.0001 for final cumulative egg count). (D) *Gr63a* mutant flies (solid green line) exhibited higher resistance to 10 mM paraquat feeding than controls (dashed red lines; *p* = 1×10^−6^). Paraquat (10 mM) in a 5% sucrose solution was fed to the flies. (E) *Gr63a^1^* mutants exhibited normal daily activity patterns (left), overall activity, and overall sleep (right). Activity data are representative of at least three independent experiments using Trikinetics activity monitoring hardware. Each trace is the composite average of 5 d of recording and 16+ animals per group. General trends were confirmed using video-based analysis. Error bars represent standard errors of the mean. ZT, zeitgeber.

We first asked whether the *Gr63a^1^* mutation affects general physiology. *Gr63a^1^* mutant flies were of similar size and weight as control animals and contained equivalent amounts of protein (unpublished data). Triglyceride levels in *Gr63a^1^* mutant flies, however, were significantly increased in two genetic backgrounds (Figure 3A). In observations that mirror the longevity data, flies heterozygous for *Gr63a^1^* exhibited normal levels of triglycerides (Figure S3B), and the increased fat deposition seen in homozygous mutant animals was rescued by transgenic expression of *Gr63a* (Figure 3A). Surprisingly, in spite of this increase in triglyceride storage, *Gr63a^1^* mutants did not show an increase in starvation resistance (Figures 3B). These results indicate that that *Gr63a* modulates multiple aspects of fat metabolism in *Drosophila*.

We next measured daily egg production in *Gr63a^1^* mutant females and control mutant females for 10 d early in life. Virgin females of both genotypes were collected and exposed individually to two control males (*w^1118^*). Female *Gr63a* mutants laid more eggs than the control females on each of the observed days. Although the daily differences were not always statistically significant, the cumulative number of eggs laid over the first 2 wk was significantly greater in the *Gr63a* mutants (Figure 3C). When plotted on a log-scale, it is apparent the mutant females laid proportionally greater numbers of eggs each day (roughly 40% more, Figure 3C, inset). These data suggest that enhanced longevity via *Gr63a* mutation does not require a reduction in reproductive output and may result in increased reproduction.

We next asked whether *Gr63a* mutation increased resistance to oxidative stress. In both the *w^1118^* and *yw* genetic backgrounds, *Gr63a* mutants exhibited significant resistance to 10 mM paraquat (methyl-viologen), suggesting these flies have increased oxidative stress resistance (Figures 3D and S5). Heterozygous animals were no more resistant than wild-type controls (Figure S3C), which correlates well with their similarity in longevity, fat storage, and CO_2_-sensing phenotypes.

General activity patterns and the strength of the sleep–wake cycle are affected by aging and may be considered functional measures of health in the fly [25],[26]. We therefore asked whether loss of *Gr63a* affected either of these phenotypes. Based on standard beam-crossing assays (e.g., using the Trikinetics DAM equipment), we found no evidence for biologically relevant changes in circadian activity patterns, reductions in overall activity, or changes in general sleep patterns (Figure 3E). Although *Gr63a* mutants exhibited a trend toward less sleep per day, this only reached statistical significance in one of three experiments. General activity patterns were confirmed on a subset of the Trikinetics data using video-based analysis.

### Gr63a Acts through Mechanisms Distinct from Dietary Restriction to Modulate Lifespan

**Figure 4:**
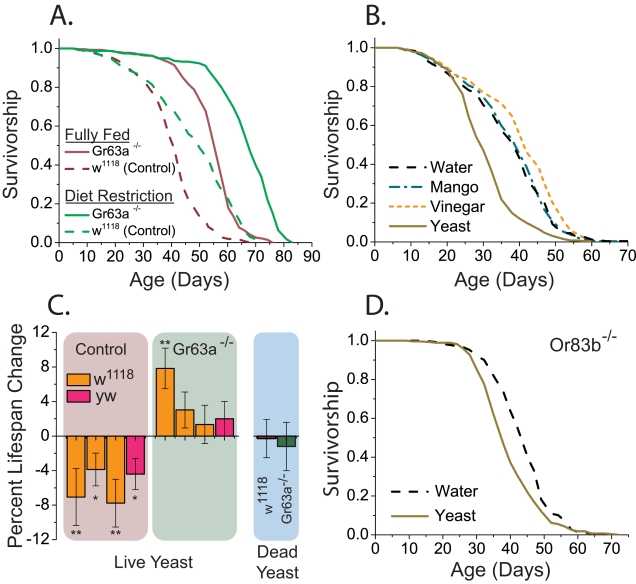
The role of Gr63a in longevity through diet restriction and exposure to food-derived odors. (A) Longevity of *Gr63a^1^* mutant flies was further extended by dietary restriction. *Gr63a* mutants live significantly longer when fed a low-nutrient diet (5% sugar-yeast diet, green solid line) than when fed a high-nutrient diet (15% sugar-yeast diet; red solid line, *p*<1×10^−15^). A similar diet-dependent extension of lifespan was observed in control animals (red vs. green dashed lines; *p* = 1×10^−10^). Sample sizes are as follows: fully fed: control, 269; mutant, 270; diet restriction: control, 251; mutant, 235. (B) Of a range of food-based odorants tested, such as mango scent and apple cider vinegar, only odors from live yeast consistently affected lifespan of *w^1118^* female flies (see Table S2 for additional details). Samples sizes are water, 258; mango, 262; vinegar, 260; and yeast, 244. (C) Yeast odors failed to impact lifespan of *Gr63a^1^* mutants. In multiple experiments involving control and mutant flies in two independent genetic backgrounds (*w^1118^* and *yw*), *Gr63a* mutants did not exhibit reduced lifespan when exposed to odor from live yeast. In all four experiments, there was a tendency for yeast odor to increase *Gr63a^1^* mutant lifespan, but the effect was small and statistically significant in only one instance. Killing of the yeast through autoclaving removes the lifespan-shortening effects in wild-type flies. (**p<0.001, *p<0.01; see also Figure S6B). (D) Flies that are homozygous for a null mutation in Or83b have greatly reduced general olfactory capabilities but are able to sense CO_2_ normally. The lifespan of Or83b animals was also reduced by live yeast odor (*p* = 1×10^−8^). Samples sizes are: control, 274; yeast-odor supplemented, 279. All odorant-supplemented longevity experiments were carried out using 5% (low nutrient) sugar-yeast medium. Error bars represent standard errors of the mean.

Dietary restriction, the reduction of nutrient uptake without malnutrition, has been shown to increase the lifespan of many species across a broad range of taxa, but the underlying causal mechanisms remain largely unknown [27],[28]. Work from invertebrate systems suggests that there is a neural basis for its effects, and neurosensory systems may play an important role [8],[9],[29],[30]. We therefore asked whether enhanced longevity through dietary restriction requires Gr63a. Adult flies of both *Gr63a* mutant and control lines were maintained in either a nutrient-replete environment or one of dietary restriction. The diet-restriction medium was chosen to maximize diet-dependent longevity extension [31],[32]. Dietary restriction further increased the longevity of *Gr63a* mutant flies (Figure 4A), and this increase was of similar magnitude to that seen in control flies. We found no evidence for differential food consumption in mutant flies (Figure S3D). These data suggest that Gr63a acts through a pathway independent of dietary restriction to impact fly lifespan.

### Gr63a Is Required for Live Yeast Odorants to Modulate Lifespan

Odorants derived from live yeast paste have been shown to affect lifespan in flies [8]. It was suggested that this complex odor altered longevity programs in response to perceived food availability because diet-restricted flies were affected by the odors, but fully fed flies were not [8]. The specific components of the yeast odor responsible for the longevity effect, however, were not identified. As an ongoing effort in our laboratory, we tested a wide range of chemically defined odorants, both attractive and aversive, and found none to have a significant effect on lifespan when presented chronically at nontoxic levels (Figure 4B, Table S2). Indeed, although we were able to repeatedly observe reduced lifespan upon exposure to live yeast odors, even highly attractive odorants that might also be considered indicative of a nutritious food source, such as mango scent [33] and apple cider vinegar, had no effect or had a modest positive effect on longevity (Figure 4B). It is therefore not sufficient for an odor to be favorable or food-related to impact *Drosophila* longevity.

Unlike our mixtures, the yeast odor that impacts fly lifespan is dynamic and generated by live organisms. We therefore decided to investigate odors that would be characteristic of live preparations. CO_2_ is released by live yeast as a byproduct of fermentation, and having observed that loss of CO_2_ sensing in *Gr63a* mutants may enhance longevity, we wondered whether CO_2_ might contribute to the longevity-limiting effect of live yeast. We first asked whether Gr63a affects the ability of flies to detect and respond to live yeast paste. We found that *Gr63a* mutant flies were significantly less successful locating yeast in a two-choice odor trap assay, suggesting that the mutants are deficient in the ability to detect the yeast, have reduced motivation to pursue it, or both (Figure S6A). When live yeast was competed against yeast that were killed by autoclaving, wild-type flies exhibited a significant preference for live yeast, whereas *Gr63a^1^* mutants were less able to distinguish between the two (*p* = 0.03; unpublished data). These results suggest that CO_2_ plays a role in the detection of live yeast paste and that Gr63a is important for chemotaxis to this food source.

If CO_2_ is an important cue for modulating longevity from yeast odorants, then live yeast should fail to impact the lifespan of *Gr63a^1^* mutant flies. Consistent with this prediction, we found that the odor of live yeast modestly reduced lifespan compared to controls in two wild-type genetic backgrounds (*w^1118^* and *yw*) and in replicate trials (Figure 4C, *p*<0.002), whereas in three out of four cases, yeast paste odorants failed to significantly impact the longevity of *Gr63a* mutant flies (Figure 4C). In the single instance where there was a statistically significant effect, exposure to the yeast odor resulted in a moderate *increase* in Gr63a lifespan. Indeed, there was a trend in all four replicates for yeast paste to increase the lifespan of flies with the *Gr63a^1^* mutation (Figure 4C). Notably, *Or83b* mutants, which are broadly anosmic but retain the ability to sense CO_2_ [34], exhibited a normal longevity response to odorants from live yeast (Figure 4D). Finally, consistent with a requirement for the yeast to be alive, we found that autoclaved yeast did not alter the lifespan of wild-type or *Gr63a^1^* mutant flies (Figure 4C, right, see also Figure S6B).

## Discussion

Evidence from work in the nematode worm, *C. elegans*, and in the fruit fly, *D. melanogaster*, has established that aging is strongly modulated by sensory systems and that this modulation is evolutionarily conserved. In the worm, ablation of specific sensory neurons increases lifespan, as do mutations in genes required for sensory signal transduction [6],[7]. In flies, exposure to food-based odorants is sufficient to reduce lifespan and partially reverse the beneficial effects of dietary restriction [8]. Furthermore, odorant receptor Or83b loss of function, which abolishes olfactory responses in a majority of olfactory neurons and leaves flies broadly anosmic, results in significantly increased lifespan. Olfactory signaling also impacts stress response and energy balance; *Or83b* mutant flies are resistant to starvation and hyperoxia, and females have gross alterations in fat deposition [8].

There are at least two possible explanations for the *Or83b* mutant phenotype. First, global loss of chemosensory function and a general lack of olfaction may be required for extended longevity. Alternatively, it may be that the longevity observed in *Or83b* mutant animals results from an inability to detect a small number of specific odorants, as identified and processed by specific odorant receptors. To distinguish these alternatives, we have begun investigating the lifespans of flies with loss of function in individual odorant receptors that are known to have ecologically important ligands.

We focused on Gr63a because of its specialized role in CO_2_ sensing. At low concentrations, CO_2_ serves as a stress pheromone and elicits avoidance behavior [15], whereas in other contexts, it may carry information about nutritious food sources [14]. Unlike Or83b, Gr63a is associated with highly specific olfactory functions. It is expressed in only a small number of specialized olfactory neurons, which are found in the ab1 sensilla of the antennae [16],[17]. These neurons send axons to a single glomerulus, the V glomerulus, in the most ventral portion of the antennal lobe of the brain, which responds selectively to CO_2_ [15]. Recent work has established that Gr63a colocalizes with a second receptor, Gr21a, and both are required for gaseous CO_2_ chemosensation [17],[18]. CO_2_ sensing is likely confined to *Gr63a*/*Gr21a*-expressing neurons; of 39 other chemosensory receptors examined, none exhibited a comparable response to CO_2_ [17].

Our results support a model in which the sensing of CO_2_ limits lifespan, fat storage, and some aspects of stress resistance. Flies homozygous for a null allele of *Gr63a* (*Gr63a^1^*) are long-lived, fat, and resistant to the oxidative stress–generating compound paraquat. Ablation of *Gr63a*-expressing cells phenocopies the mutant longevity phenotype. Furthermore, the Gr63a receptor is required for sensory perception of live yeast to have an influence on fly lifespan. Together, these data implicate a highly specialized neurosensory circuit involving CO_2_ detection and subsequent activation of ab1C neurons and the V glomerulus as sufficient to modulate fly lifespan and physiology.

Why does perception of CO_2_ lead to changes in lifespan? One possibility is that it is an important component of complex, food-based smells and provides information about nutrient availability. Faucher et al. postulated that CO_2_ plays a role in the behavioral response to profitable food sources [14], and certain food sources that emit CO_2_ also emit odorants that reduce CO_2_ sensitivity and inhibit avoidance behaviors [35]. Compared to control animals, *Gr63a^1^* mutants were less attracted to live yeast. It must be kept in mind, however, that *Gr63a* mutant animals exhibited enhanced fat storage. This difference in energy reserves may translate into reduced motivation to locate food in our odor trap assay in which flies become increasingly food deprived. Nevertheless, wild-type flies are less attracted to killed yeast than they are to live yeast, which provides further support for this idea (unpublished data). If food perception is the key to the longevity effect, then it is somewhat perplexing that lifespan extension through Gr63a acts additively with standard diet manipulation. A second possibility derives from the suggested role of CO_2_ as a stress pheromone [15]. In our aging experiments, flies are kept together as small groups in vials where it is conceivable that CO_2_ levels are slightly elevated compared to ambient air. Perhaps *Gr63a* mutant flies experience less chronic stress and are healthier as a result. This explanation is unlikely. We measured CO_2_ levels in the vials and were unable to detect a significant difference from normal room air (unpublished data). We also observed similar increases in Gr63a lifespan when flies were maintained at lower densities (unpublished data). Further analysis of the molecular and physiological changes induced by CO_2_ sensing or Gr63a loss of function may help distinguish these alternatives.

Perhaps some understanding of the influence of CO_2_ in *Drosophila* can be gleaned from its sex-specific effects. We find that loss of function in Gr63a clearly affects female lifespan but not the lifespan of males. Sex-specific effects on lifespan in *Drosophila* have been reported for dietary restriction [36] and insulin signaling [37],[38] (with females more affected), and more recently for ecdysone signaling [39] (with males more affected). Overall, however, sexual dimorphisms in the response to longevity-extending manipulations have not been explored, and there is very little understanding of the underlying mechanisms [40]. Particularly relevant for this work, it was recently shown that female flies exhibit a different behavioral sensitivity to CO_2_ compared to males. The presence of food-related odors, for example, sensitizes females, but not males, to avoid low concentrations of the gas [14]. This may be related to physiological requirements; female flies require high protein–based food sources to support egg laying. Consistent with this interpretation, we found that male lifespan was unaffected by *Gr63a* mutation or the odor of live yeast paste. The sex-specific effects of Gr63a on lifespan may therefore be due to differences in olfactory stimulation or in the processing of odor information or both.

There is growing evidence that different odorants, and their cognate odorant receptors, may be capable of modulating lifespan in different ways and with different effects [7],[29]. Although the data are not conclusive, our results suggest that food-based odors may have both lifespan-extending and lifespan-shortening components. Unlike control animals, *Gr63a* mutant flies tended to exhibit an increased lifespan in the presence of live yeast odor. This effect was always small, but it reached statistical significance in one of four experiments. Wild-type flies sensing apple cider vinegar, another food-based odorant, had a small but significantly increased lifespan compared to those exposed to a pure water control (Figure 4B, Table S1). Therefore, although our results suggest that CO_2_ is the dominant component of yeast odor that limits fly lifespan, there are indications of other odorants with beneficial effects.

Do the two olfactory receptors that impact fly lifespan, Or83b and Gr63a, impinge on the same molecular pathways to affect aging? Both mutants have similar effects on fat deposition (they increase it) and on some aspects of stress resistance (both are resistant to particular forms of oxidative stress). Both lack obvious defects in growth, general activity, or reproduction. Nevertheless, several lines of evidence point to their acting through at least partly distinct signaling pathways. First, *Or83b* mutants are broadly anosmic, but their CO_2_-sensing capabilities are largely intact [34],[41]. This is the opposite of *Gr63a* mutants, whose functional deficits are thought to be confined to CO_2_ sensing. Second, *Or83b* mutant flies exhibit enhanced resistance to a broad spectrum of environmental stressors, whereas *Gr63a* mutants are resistant to only select stresses (paraquat, but not starvation, for example). Third, longevity extension through loss of Or83b function is observed in both sexes and is partly dependent on diet. *Gr63a* mutation affects female longevity only, and we find no evidence for an interaction between dietary restriction and *Gr63a* mutation with respect to longevity extension. These considerations suggest the presence of at least two distinct neurosensory signaling pathways that act through independent neurosensory circuits to modulate fly aging.

We postulate that although different species may have distinct abilities to detect and characterize odors, the biological impact of the perceived information may be evolutionarily conserved and important for human biology [42]. For us, it may not be the smell of yeast, for example, or the sensing of CO_2_ that affects aging, but it may be the perception of food or danger. For example, the human cephalic phase response, which is elicited by the sight, smell, and taste of food, stimulates the release of hormones, including insulin and glucagon, that have been linked to aging in several model systems [29]. If true, such a model lends itself to intriguing possibilities. Sensory systems, because of their peripheral nature, are ideal targets for intervention. An incisive combination involving targeted knockdown of certain sensory inputs and stimulation of others, either through gene therapy or controlled perceptual experience, might form the basis of a simple, yet powerful, program of disease prevention and healthy aging.

## Materials and Methods

### Fly Strains

All fly stocks were obtained from the Bloomington Stock center. The stock numbers are: *Gr63a^1^* (9941), UAS-reaper (5824), UAS-*Gr63a* (23143), and *Gr63a*-GAL4 (9943). All fly stocks were backcrossed to control line(s) at least seven times to control for genomic variation.

### Lifespan Studies

To control the density of developing larvae and synchronize the emergence of adults, an equal volume of eggs was distributed into bottles containing 30 ml of cornmeal-sugar-yeast food (see recipes below in Materials and Methods and in Table S3). To ensure a tight cohort of same-age flies at the onset of the experiment, adults were collected within 24 h of eclosion and transferred to 10% sugar-yeast food for mating. On day 3, flies were then sorted under light CO_2_ anesthesia (flies were anesthetized for 3–5 min under 100% CO_2_ flowing at 5 l/min through a Flow Buddy and standard fly pushing pad from FlyStuff.com). At least eight replicate vials of 30 flies (males or females) were established for each genotype/treatment. Unless otherwise noted, all longevity experiments were performed in high nutrient conditions (15% SY medium, see below). In certain cases, for example in experiments examining lifespan of heterozygous animals, heterozygous mutant parents were crossed, and all experimental flies developed together as larvae. Adults were then treated as described above, and genotypes were sorted by eye color. Flies were transferred to fresh medium every other day, at which time dead flies were removed and recorded. Flies were kept in constant temperature (25°C) and humidity (60%) with 12∶12 light:dark cycle.

### Quantitative PCR

Flies were staged, collected, and sexed according to the procedures outlined above for lifespan assays. On day 10 posteclosion, total RNA was extracted using Trizol from Invitrogen. For each genotype/treatment, at least three independent extractions were performed using 40 fly heads for each extraction. Extracted RNA was treated with 1 U DNAse I from Invitrogen and then reverse-transcribed using Superscript III First-Strand Synthesis kit, also from Invitrogen. Real-time PCR was performed using RT^2^ SYBR green/Rox PCR master mix from SA Biosciences and an ABI 7000. The following primers were used: Gr63aF (AATTCCGGACACAGTCTCTC), Gr63aR (ATTAGCACTGTTCAGCGGTT), RP49F (ACTCAATGGATACTGCCAG), and RP49R (CAAGGTGTCCCACTAATGCAT).

### Triglyceride Assay

Flies were collected and sorted according to the procedures outlined above for lifespan assays. For each measure, five females were collected on day 10 posteclosion and were homogenized in 300 µl of PBS/0.05% Triton-X. Fly homogenate (10 µl) was then added to 200 µl of Infinity Triglyceride Reagent (Thermo Electron Corp) and incubated at 37°C for 15 min with constant agitation. Triglyceride levels were determined by measuring absorbance at 520 nm and total amounts were determined using a triglyceride standard. Data were normalized to protein levels measured via Bradford assay. Each data point was based on six replicates from three different vials.

### Odor Trap Assays

Flies were collected and sorted according to the procedures outlined above for lifespan assays. Following mating, 3-d-old females were separated from males using light CO_2_ anesthesia and placed into vials (30 flies per vial) containing 10% sugar-yeast food. Flies were transferred to new vials every other day (without anesthesia) for 7 d. The evening of the ninth day, females were place in fresh vials containing only 1% agar for overnight food deprivation. On the morning of day 10, multiple cages were established with 30 females. Each chamber contained two traps: one yeast trap and one control trap (water). Flies were monitored continuously, and the number of flies in each trap was recorded every 30 min. These data were used to calculate an attraction index (AI), which is computed as the (# of flies in yeast trap − # of flies in control trap)/(total # of flies). Flies that failed to enter one of the traps after 24 h were omitted from the total. For assays comparing autoclaved versus live yeast, the traps consisted of one live yeast trap and one trap with yeast paste that had been autoclaved on a 30-min cycle.

### T-Maze Test for CO_2_ Avoidance

Flies were collected and sorted according to the procedures outlined above for lifespan assays. Following mating, 3-d-old females were separated from males under light CO_2_ anesthesia and placed into vials (40 flies per vial) containing 10% sugar-yeast food. Flies were transferred to new vials every other day (without anesthesia) for seven days. On day 10, flies were collected without anesthesia and subjected to a T-maze. For each measure, 40 females were placed into a T-maze with ∼4% CO_2_ air stream on one side and room air on the other. Flies were given 1 min in the absence of light to decide between the two sides. For each measure, an attraction index was calculated as the (# of flies moving toward CO_2_ − # of flies moving away)/(total # of flies). Flies that failed to make a choice were discarded. At least eight replicate measures were collected for each genotype/treatment.

### Behavioral Monitoring

For most experiments, we used the DAM5 activity monitoring system (Trikinetics) for recording photobeam crosses by individual flies. Summary and statistical analyses were conducted using custom scripts with the R statistical computing platform. Flies were between 10–20 d old and monitored on 10% SY food. At least 16 flies were used per group, and locations were randomized to avoid position effects. Data were collected in 1-min bins for 5–6 d; the first day was discarded to avoid effects of CO_2_ anesthesia and adjustment to the environment. A subset of the Trikinetics data were verified using a continuous video-based monitoring system. For these experiments, video cameras were suspended above DAM5 monitors, and flies were recorded continuously for 5 d. Video analysis software developed in our laboratory was used to confirm the Trikinetics data by tracking individual animals and calculating movement frequency, distance traveled, sleep time, sleep position, and a variety of other measures. For all experiments, light, temperature, and humidity were maintained at 12-h light:12-h dark, 25°C, and 60%, respectively. Flies with >5 min of consecutive inactivity were considered to be in sleep.

### Egg Laying/Fecundity

For each genotype/treatment, 13–15 virgin female flies were collected and placed individually into vials with 15% sugar-yeast food. After 3 d, two control males were placed into each vial. Flies were transferred to fresh vials every day, and eggs were counted daily for 2 wk. Flies that did not survive the experiment were omitted.

### Oxidative Stress (Paraquat)

Flies were collected and sorted according to the procedures outlined above for lifespan assays. Following mating, 3-d-old females were separated from males under light CO_2_ anesthesia and placed into vials (20 flies per vial) containing 10% sugar-yeast food. Flies were transferred to new vials every other day (without anesthesia) for 7 d. On the evening of the ninth day, females were placed in fresh vials containing 1% agar for overnight food deprivation. On the morning of day 10, flies were transferred into vials containing 10 mM methylviologen (Invitrogen) dissolved in 5% sucrose dispensed onto filter paper. Survivorship was determined at least twice per day.

### Starvation

Flies were collected and sorted according to the procedures outlined above for lifespan assays. Following mating, 3-d-old females were separated from males under light CO_2_ anesthesia and placed into vials (20 flies per vial) containing 10% sugar-yeast food. Flies were then transferred to new vials every other day (without anesthesia) for 7 d. The morning of the tenth day, females were placed in fresh vials containing 1% agar. Survivorship was determined at least once per day.

### Odor Longevity Experiments

Flies were collected and sorted according to the procedures outlined above for lifespan assays. Flies were placed into vials as for standard longevity, but the vials were then placed into closed acrylic chambers where air was streamed into the chambers at the rate of 0.5 l per min using an Air Delivery System from ARS, Inc. (http://www.ars-fla.com/Mainpages/AirDelivery/ADS_systems.html). To insure proper airflow and to prevent air pressure changes, air left the chamber via an outlet on the opposite side. In each case, air was pumped through a particular solution prior to entering the chamber. This solution contained water-based mixtures with the appropriate odorant or vehicle control. For yeast odorants, live yeast pellets (Fleischmann's active dry) were added to water, and these solutions were changed daily. Other solutions were changed as needed. Instead of normal vial plugs, experimental vials had specially designed wire-mesh tops to allow free flow of air and odorants to the flies. The flies were transferred to fresh medium every 2–3 d, and the dead flies were removed and recorded. Low-nutrient medium (5% SY, see below) was used to maximize the longevity effect [8]. Flies were kept in constant temperature (25°C) and humidity (60%) with 12∶12 light:dark cycle. The scents described in the experiment were: (1) mango scent: ethanol, acetic acid, and 2-phenylethanol were mixed in a ratio of 1∶22∶5. This mixture was then mixed 1∶99 with water. See [33] for additional details; (2) apple cider vinegar: 10% store-bought apple cider vinegar mixed in water; (3) yeast paste: 5 g of live bakers' yeast added to 50 ml of water; (4) dead yeast paste: live yeast were autoclaved for 30-min gravity cycle, and then used at 5 g/50 ml as for live yeast paste; (5) ethanol: 3% ethanol in water; (6) hexanol: 1% hexanol dissolved in paraffin oil; and (7) isoamyl acetate: 1% isoamyl acetate in paraffin oil.

### Feeding Assay

Flies were collected and sorted according to the procedures outlined above for lifespan assays. Fifteen-day-old female flies were placed on 10% sugar-yeast food containing FD&C Blue #1 for 6 h beginning at noon. Each vial containing five flies was homogenized in 75 µl of PBS +0.05% Triton X-100 using a TissueLyser bead mill (Qiagen) and filtered to remove large particles. Absorbance at 620 nm was measured and compared to a standard curve. Dye levels were normalized to total protein as measured by the bicinchoninic acid (BCA) method (Pierce). Each column represents the data from eight different vials per group.

### Statistical Analysis

Unless otherwise indicated, pairwise comparisons between control and mutant survivorship curves were carried out using the statistical package R with WinChecker, a survival analysis package developed in the Pletcher Laboratory. *p*-Values were obtained using log-rank test. For comparisons involving gene expression, pairwise *t*-tests were carried using independent RNA extractions as the unit of observation. Comparisons between mutant and control lines for triglyceride levels and egg production were obtained using *t*-test with vial (or group of flies) as the observational unit, whereas activity measures were compared from individual flies.

### Fly Food

Please refer to Table S3 for the ingredients for each of the diets used in this paper. Water (1) and agar were combined in a large kettle. The solution was simmered under slow mixing for 40 min. We then combined water (2), yeast, sucrose, dextrose (MP Biomedicals), and cornmeal (SYSCO Corp.) in a separate container and mixed well. This mixture was then added to the agar solution, and mixing speed was increased while the food boiled for 15 min. Heat was removed, and the food was allowed to cool to 65°C, after which, tegosept and propionic acid were added, and the food was dispensed into 150-ml bottles or 28.5×95-mm vials (Genesee Scientific).

## Supporting Information

Figure S1 **Verification of** ***Gr63a^1^*** **mutant deficiency in CO_2_ sensing.** Flies were placed in a T-maze for 1 min without light. One end of the T-maze contained air, and one end contained ∼4% CO_2_. Flies that did not enter either chamber were discarded. Forty flies were used for each measure, and a minimum of eight measures were made for each genotype.
(0.47 MB EPS)

Figure S2 **Details of female-specific longevity extension in** ***Gr63a^1^*** **mutant flies.** (A) Longevity extension in *Gr63a* homozygous mutant flies is female specific. After 12 generations of backcrossing, Gr63a male longevity was indistinguishable from control males (*p* = 0.19, log-rank test). *n* =  control, 224; mutant, 197. (B) Longevity extension in *Gr63a* mutant females persisted after 20 generations of backcrossing (*p* = 2×10^−7^). *n* =  control, 228; mutant, 243. (C) Age-specific mortality rates for *Gr63a* homozygous mutant and control females. Age-specific mortality is calculated as −ln(px) where px is the probably of surviving age x. Mortality rates are presented on the natural log scale.
(1.05 MB EPS)

Figure S3 **Flies heterozygous for the** ***Gr63a^1^*** **exhibit no obvious phenotypes.** (A) Flies carrying only a single copy of the *Gr63a^1^* null allele exhibited lifespan similar to that seen in wild-type flies (*p* = 0.24 comparing heterozygotes to wild-type). *n* =  control, 220; heterozygous mutants, 231, homozygous mutant, 213. (B) *Gr63a^1^* heterozygotes have similar levels of triglyceride that are statistically indistinguishable from controls (*p* = 0.24) but significantly different from *Gr63a* homozygous mutants (*p* = 0.03). (C) *Gr63a^1^* heterozygous flies have normal resistance to paraquat (*p* = 0.73). (D) *Gr63a^1^* mutant flies consume food at the same rate as wild-type animals (*p* = 0.52). Food consumption is expressed in terms of micrograms of blue dye ingested per microgram of fly protein, over a 6-h period.
(1.11 MB EPS)

Figure S4 **Age-specific mortality rates for flies expressing the proapoptotic gene reaper using** ***GR63a*** **-GAL4 to ablate the associated neurons.** Female *Gr63a*>reaper flies exhibited consistently lower mortality rate than corresponding controls, including *w^1118^* wild type, UAS-reaper only, and *Gr63a*-Gal4-only. Age-specific mortality was calculated as −ln(px) where px is the probably of surviving age *x*. Mortality rates are presented on the natural log scale.
(0.62 MB EPS)

Figure S5 ***Gr63a^1^*** **homozygous flies in the** ***yw*** **genetic background exhibited higher resistance to oxidative resistance paraquat.** After backcrossing for ten generations, *Gr63a* mutants show enhanced resistance to oxidative damage when placed on 10 mM paraquat (*p* = 9×10^−12^).
(0.51 MB EPS)

Figure S6 **Behavioral and longevity responses to live yeast paste in** ***Gr63a^1^*** **mutants.** (A) *Gr63a* flies are less attracted to odor from live yeast paste. For each measure, 30 females were placed in a chamber containing one yeast trap and one control trap (water). Flies were counted at 30-min time intervals. At least four replicate measures were obtained for each genotype. Attraction index is calculates as (# of flies in yeast trap − # of flies in control trap)/(total # of flies). Flies that did not enter one of the traps overnight were omitted. (B) Yeast paste odorants do not affect Gr63a longevity. One representative experiment (of those shown in Figure 4C) showing the survivorship data for *Gr63a* mutants, whose lifespan is unaffected by exposure to live yeast–based odorants (*p* = 0.86). Autoclaved yeast does not affect the lifespan of either wild-type or *Gr63a* homozygous mutant flies. (*p* = 0.4 and 0.3, respectively). *n* =  mutant flies: dead yeast, 228; live yeast, 211; water, 233. Control flies: dead yeast, 235; live yeast, 231; water, 227.
(0.97 MB EPS)

Table S1 **Summary of** ***Gr63a*** **mutant longevity data.**
(0.02 MB XLS)

Table S2 **Summary of odor-longevity data.**
(0.04 MB DOC)

Table S3 **Fly media ingredients.**
(0.04 MB DOC)
