# Supplementary material for: Carbon Dioxide Sensing Modulates Lifespan and Physiology in Drosophila
Source: PLoS Biol. 2010 Apr 20;8(4):e1000356. doi: 10.1371/journal.pbio.1000356 (PMC2857880; doi:10.1371/journal.pbio.1000356)
Supplement: Table S2 — Summary of odor-longevity data. (0.04 MB DOC) [file pbio.1000356.s008.doc]

***Table S2. Summary of experiments testing the impact of specific odors on fly lifespan.*** Only yeast odors had a consistently significant effect across genotypes. Flies exposed to vinegar odors exhibited an increased lifespan, but the mechanism is somewhat mysterious as Or83b mutant flies are expected to have a severely diminished capacity to smell vinegar, yet they have a comparable lifespan extension when exposed to the odor. It was also recently shown that Hexanol can inhibit ab1C neurons and reduce CO_2_ sensing [35], but we do not find a significant effect on lifespan.

| **Genotype** | **Odor Solvent** | **Ligand** | **% change** | **P-value** |
| --- | --- | --- | --- | --- |
| Canton-S | Water | Yeast | -10.0 | 3.2x10^-6^ |
|  |  | Vinegar | +0.8 | 0.68 |
|  |  | Ethanol | -6.8 | 0.00054 |
|  | Paraffin oil | Hexanol | +0.4 | 0.84 |
|  |  | Isoamyl acetate | +2.9 | 0.27 |
| Or83b | Water | Yeast | -9.2 | 1.9 x10^-6^ |
|  |  | Vinegar | +7.3 | 2.7x10^-5^ |
|  |  | Mango | -1.4 | 0.11 |
|  |  | Ethanol | +4.0 | 0.011 |
|  | Paraffin oil | Hexanol | +0.7 | 0.93 |
|  |  | Isoamyl acetate | -0.5 | 0.73 |
| W1118 | Water | Yeast | -17.0 | 6.2x10^-14^ |
|  |  | Vinegar | +8.4 | 0.0022 |
|  |  | Mango | +1.8 | 0.86 |
|  |  | Ethanol | +6.7 | 0.0051 |
|  | Paraffin oil | Hexanol | +2.0 | 0.69 |
|  |  | Isoamyl acetate | +1.5 | 0.53 |
