# Supplementary material for: Carbon Dioxide Sensing Modulates Lifespan and Physiology in Drosophila
Source: PLoS Biol. 2010 Apr 20;8(4):e1000356. doi: 10.1371/journal.pbio.1000356 (PMC2857880; doi:10.1371/journal.pbio.1000356)
Supplement: Table S3 — Fly media ingredients. (0.04 MB DOC) [file pbio.1000356.s009.doc]

| **Table S3: Fly media ingredients** | | | | | |
| --- | --- | --- | --- | --- | --- |
|  | **Larval Media** | **Mating Media** | **5% SY** | **10% SY** | **15% SY** |
| **Component** | **Amount** | **Amount** | **Amount** | **Amount** | **Amount** |
| Water (1) | 800 ml | 750 ml | 750 ml | 750 ml | 750 ml |
| Water (2) | 200 ml | 250 ml | 250 ml | 250 ml | 250 ml |
| Agar | 10 g | 20 g | 21 g | 21 g | 21 g |
| Dextrose | 55 g | - | - | - | - |
| Corn Meal | 60 g | - | - | - | - |
| Sucrose | 30 g | 100 g | 50 g | 100 g | 150 g |
| Yeast | 25 g | 100 g | 50 g | 100 g | 150 g |
| 20% Tegosept | 15 ml | 15 ml | 15 ml | 15 ml | 15 ml |
| Propionic Acid | 3 ml | 3 ml | 3 ml | 3 ml | 3 ml |
